# Preparation and Characterization of Chitosan-Coated Manganese-Ferrite Nanoparticles Conjugated with Laccase for Environmental Bioremediation

**DOI:** 10.3390/polym13091453

**Published:** 2021-04-30

**Authors:** Azzurra Apriceno, Ilaria Silvestro, Annamaria Girelli, Iolanda Francolini, Loris Pietrelli, Antonella Piozzi

**Affiliations:** 1 Department of Chemistry, Sapienza University of Rome, P.le Aldo Moro 5, 00185 Rome, Italy; aapriceno@ibecbarcelona.eu (A.A.); ilaria.silvestro@uniroma1.it (I.S.); annamaria.girelli@uniroma1.it (A.G.); iolanda.francolini@uniroma1.it (I.F.); loris.pietrelli@uniroma1.it (L.P.); 2 Insitute for Bioengineering of Catalonia, Parc Científic de Barcelona (PCB), c/Baldiri Reixac 10-12, 08028 Barcelona, Spain

**Keywords:** laccase, magnetic nanoparticles, chitosan, immobilized enzyme, bioremediation, diclofenac removal

## Abstract

Bioremediation with immobilized enzymes has several advantages, such as the enhancement of selectivity, activity, and stability of biocatalysts, as well as enzyme reusability. Laccase has proven to be a good candidate for the removal of a wide range of contaminants. In this study, naked or modified MnFe_2_O_4_ magnetic nanoparticles (MNPs) were used as supports for the immobilization of laccase from *Trametes versicolor*. To increase enzyme loading and stability, MNPs were coated with chitosan both after the MNP synthesis (MNPs-CS) and during their formation (MNPs-CS_in situ_). SEM analysis showed different sizes for the two coated systems, 20 nm and 10 nm for MNPs-CS and MNPs-CS_in situ_, respectively. After covalent immobilization of laccase by glutaraldehyde, the MNPs-CS_in situ_-*lac* and MNPs-CS-*lac* systems showed a good resistance to temperature denaturation and storage stability. The most promising system for use in repeated batches was MNPs-CS_in situ_-*lac*, which degraded about 80% of diclofenac compared to 70% of the free enzyme. The obtained results demonstrated that the MnFe_2_O_4_-CS_in situ_ system could be an excellent candidate for the removal of contaminants.

## 1. Introduction

The use of enzymes in free form is generally associated with some limitations, such as high cost of production and purification, poor stability in a large range of pH and temperature, difficult recovery, and no reuse of the catalyst. Enzyme immobilization onto or within solid carriers is a way to overcome these shortcomings. Indeed, highly performant biocatalysts with better temperature and pH stability as well as with high catalytic activity, if compared to free proteins, can be obtained by using immobilization techniques [1]. The simplest method to immobilize enzymes onto surfaces is based on physical adsorption. This method takes advantage of being reversible, having low cost, and allowing the reuse of the support after enzyme inactivation. However, because physical adsorption has the inconvenience of potential enzyme release into the reaction medium, covalent binding is usually preferred. In this latter case, generally, crosslinking agents such as glutaraldehyde or epichlorohydrin are used [2].

Laccase (E.C. 1.10.3.2, p-benzenediol: oxygen oxidoreductase) is an enzyme able to catalyze the oxidation of various aromatic and non-aromatic compounds with the reduction of molecular oxygen to water [3]. Laccases are widely distributed in plants, fungi, bacteria, and insects. Fungal laccases possess a redox potential higher than that of plants or bacteria; therefore, they are implicated in several biotechnological applications, especially in the degradation of lignin, in the food industry, and environmental bioremediation [4,5,6,7].

Immobilization of various types of laccases with a wide range of different methods and supports has been studied over the years [8]. Several investigations have shown that inorganic materials, such as alumina silicates, titania, and other oxides, are excellent supports for enzyme immobilization thanks to their high thermal and mechanical resistance, as well as to the presence of functional groups on the surface that can bind the biocatalyst [2,9,10]. Recently, the use of magnetic nanoparticles (MNPs), for example, iron oxides, as supports for enzyme immobilization has increased [11]. These matrices, owing to their small size (high surface to volume ratio), reduce troubles related to low mass transfer and allow the enhancement of protein loading. In addition, by applying an external magnetic field, it is possible to recover the biocatalyst easily and efficiently from the reaction medium, thus obtaining no contamination of final products and repeated use of the enzyme.

Biological treatments (bioremediation) exploiting enzymatic catalysis are considered a valid alternative to classical chemical or physical methods for the degradation of pollutant compounds [12]. Indeed, bioremediation with enzymes has several advantages such as the reduced impact on ecosystems as well as selectivity, regioselectivity, and stereospecificity for compounds of interest. Many contemporary pollutants come from pharmaceutical, cosmeceutical, biomedical, and personal care products utilized by people. Diclofenac (DFC) is one of the most common contaminants found in the aquatic environment, belonging to the category of non-steroidal anti-inflammatory drugs (NSAIDs) [13,14,15]. The presence of these unconventional contaminants (UCs) in surface water and wastewater is an issue of considerable scientific interest for their possible toxic effects [16]. Indeed, UCs can provoke damage to the nervous, hormonal, and reproductive systems of aquatic and non-aquatic organisms, as well as of fauna. These contaminants, classified as emerging compounds, still have no regulatory standards or limitations. The conventional wastewater treatment methods are often ineffective and therefore specific processing is necessary for the removal of these UCs.

In the present work, manganese iron oxides nanoparticles were synthesized by a reverse micro-emulsion method and then used as a support for the covalent immobilization of laccase from *Trametes versicolor* for the degradation of environmental unconventional pollutants. To introduce functional groups on magnetic nanoparticle surface able to bind the enzyme and then to enhance the stability of immobilized enzyme, chitosan (CS) was chosen as a coating material by employing two different methods (during and after nanoparticle synthesis). CS is a naturally occurring polysaccharide, biocompatible, biodegradable, non-toxic, with antibacterial and antioxidant features, possessing amino and hydroxyl groups [17,18]. The nanoparticles were characterized by infrared spectroscopy, field emission scanning electron microscope observations (FESEM), and elemental analysis.

Laccase was covalently immobilized onto functionalized MNPs by using various enzyme concentrations. The catalytic activity was assessed with 2,2′-azino-bis-(3-ethyl-benzothiazoline-6-sulfonic acid) (ABTS).

In addition, to reduce the surface free energy and to avoid nanoparticle aggregation, surfactants are generally used for MNPs synthesis. In this work, sodium dodecylbenzenesulfate (NaDBS) was employed in the MNP preparation. To compare the influence on nanoparticle properties of a polymer stabilizing with respect to that of a surfactant, MNPs were synthesized replacing NaDBs (sodium dodecylbenzenesulfate) with chitosan, thus avoiding the subsequent coating step required for enzyme immobilization. Activity, stability, and reusability of all the obtained systems at different pH and temperature were studied and compared with the characteristics of free enzymes. Finally, the systems containing the immobilized biocatalyst were employed in the degradation of diclofenac, chosen as a model pharmaceutical pollutant.

## 2. Experimental Procedure

### 2.1. Materials

Laccase from *Trametes versicolor* (specific activity = 136 U/mg), chitosan (CS, Mn = 280,000 g/mole, viscosity 200–800 cP at 25 °C), sodium dodecylbenzenesulfonate (NaDBS), 2,2′-Azino-bis(3-ethylbenzothiazoline-6-sulfonic acid) diammonium salt (ABTS), acetic acid, glutaraldehyde (50% solution in water) and Bradford reagent were purchased from Sigma Aldrich. Iron nitrate nonahydrate (FeN_3_O_9_·9H_2_O) and manganese nitrate tetrahydrate (Mn(NO_3_)_2_·4H_2_O) were purchased from Riedel-de Haën. All reagents employed were of high purity. When necessary, the solvents were degassed under nitrogen flow. All used solutions were prepared in double distilled water.

### 2.2. Magnetic Nanoparticle Synthesis

Synthesis of manganese iron oxide nanoparticles (MnFe_2_O_4_) was carried out, employing a water in toluene microemulsion procedure, as previously reported [19].

Briefly, FeN_3_O_9_·9H_2_O and Mn(NO_3_)_2_·4H_2_O were dissolved in distilled water (11.5 mL) at final Fe^3+^ and Mn^2+^ concentrations of 0.4 M and 0.2 M, respectively. Then, a 0.4 M solution (12.5 mL) of sodium dodecylbenzenesulfate (NaDBS), previously homogenized for 15 min, was added together with excess toluene (1:20 water:toluene volume ratio). The obtained emulsion was kept for 24 h under stirring and N_2_ atmosphere to avoid Mn^2+^ oxidation. Finally, 20 mL of 1 M NaOH solution was added. The microemulsion, left under stirring for 2 h, was then kept at 100 °C for 90 min in a silicone oil bath (digestion process) under nitrogen flow. The precipitated powder (magnetic nanoparticles, MNPs) was collected by centrifugation and repeatedly washed with a 1:1 water:ethanol solution. After the purification step, MNPs were first dried at 40 °C and then submitted to a thermal treatment of calcination in an oven at 600 °C for 15 min.

### 2.3. MNP Coating with Chitosan

Magnetic nanoparticle polymer coating was carried out by two different methods: (1) CS-coating after MNP formation (CS coating); and (2) CS-coating during MNP synthesis (CS in situ). Method 1 (CS coating): 20 mg of MNPs were suspended in water (1 mL) by sonication for 10 min. Then, 1 mL of acid acetic aqueous solution (1% *v*/*v*) containing 4 mg of CS (5:1 MNPs:CS ratio) was added. After 30 min, in order to precipitate the polymer onto MNPs and restore CS amino groups, 5 mL of 1 M NaOH was added to the suspension. The coated nanoparticles were recovered by centrifugation, washed with water and THF to eliminate excess polymer, and dried in oven at 55 °C for 2 h. This nanocomposite was named MNPs-CS.

Method 2 (CS in situ): during MNP synthesis, performed as reported in Section 2.2, NaDBS was replaced with a CS acidic solution at 0.4 M concentration. MNPs were precipitated and washed as described above. The calcination process was carried out at 250 °C for 3 h instead of 600 °C for 15 min, to avoid polymer degradation. This nanocomposite was called MNPs-CS_in situ_.

### 2.4. Characterization of MNPs before and after Chitosan Coating

#### 2.4.1. Elemental Analysis

Chemical compositions of pristine and CS-coated MNPs were determined by a Carlo Erba instrument EA 1110 CHNS-O elemental analyzer (Carlo Erba Reagents SAS, Chaussée du Vexin, France). Content of the polymer coating in each nanocomposite was determined by the ratio of C content of the CS-coated MNPs and that of the pristine polymer.

#### 2.4.2. Fourier-Transform Infrared Spectroscopy—Attenuated Total Reflectance (FTIR–ATR) Analysis

Infrared analysis in attenuated total reflection mode (IR-ATR) was performed by using a Thermo Nicolet 6700 instrument equipped with a Golden Gate diamond single reflection system ((Thermo Fisher Scientific, Waltham, MA, USA). Spectra were recorded in the range 4000–650 cm^−1^, acquiring 200 scans at a resolution of 2 cm^−1^.

#### 2.4.3. Morphological Analysis

Morphological analysis of pristine and CS-coated MNPs was carried out by employing a high-resolution field emission scanning electron microscope (HR-FESEM, AURIGA Carl Zeiss AG, Oberkochen, Germany) combined with a Bruker X-ray Energy Dispersive Spectroscopy (EDS). This latter technique was also used to verify the Mn:Fe ratio in the MnFe_2_O_4_ sample.

### 2.5. Laccase Immobilization onto Chitosan-Coated MNPs

Laccase immobilization onto chitosan-coated nanoparticles was performed by the support activation with glutaraldehyde. Briefly, the nanocomposite (20 mg) was put in contact with 1 mL of glutaraldehyde (1% *v*/*v* in 0.05 M phosphate buffer pH = 7.0) at 0 °C and under continuous stirring. After 3 h, MNPs were filtered and washed with distilled water in order to remove the excess of bifunctional agent. Then, a laccase solution (0.9 mL), at different concentrations, ranging from 0.20 to 1.0 mg/mL, was added to the activated nanocomposite (20 mg), and the reaction was carried out at 4 °C for 5 h, under continuous stirring. MNPs were separated from the reaction medium by a magnet. Finally, MNPs were washed with a phosphate buffer solution to remove the physically adsorbed laccase and stored at 4 °C before use.

For comparison, a physical adsorption of laccase onto MNPs, in the absence of glutaraldehyde, was also performed, following the procedure reported above and employing an enzyme concentration of 0.20 mg/mL, which resulted to be the most suitable for enzyme immobilization.

### 2.6. Laccase Immobilization Yield

The immobilization yield was expressed as the protein binding efficiency. The protein concentration was determined by using the modified Bradford assay [20]. Particularly, the enzyme concentrations in the solutions, initial solutions, plus those deriving from washings, were estimated from a calibration curve obtained by employing laccase as a standard protein. Briefly, 100 μL of a laccase solution containing different amounts of protein (3.7–100 μg) were added to 1 mL of the Bradford reagent and 1 mL of distilled water. The calibration curve was constructed by measuring the absorbances of the Coomassie dye–laccase complex at 620 nm, after 5 min of contact, by a UV–visible spectrophotometer.

Immobilization yield (%) was defined as:

(1) $$\mathrm{Immobilization}\mathrm{yield}=\frac{\mathrm{Amount}\mathrm{of}\mathrm{immobilized}\mathrm{protein}\;(\mathrm{mg})}{\mathrm{Initial}\mathrm{enzyme}\mathrm{amount}\;(\mathrm{mg})}\times 100$$

where the amount of immobilized enzyme was determined from differences between the amount of initial enzyme and the amount remaining in the solution after immobilization, plus that in washing solutions [21].

The amount of immobilized enzyme was also determined from the difference between the amount of the initial enzyme and the amount detected in washing solutions (not bonded) and normalized with respect to the initial support weight (immobilization efficiency immobilized laccase):

(2) $$\mathrm{Immobilized}\mathrm{laccase}=\frac{\mathrm{Amount}\mathrm{of}\mathrm{immobilized}\mathrm{protein}\;(\mathrm{mg})}{\mathrm{Weight}\mathrm{of}\mathrm{support}\;(g)}$$

### 2.7. Free and Immobilized Laccase Activity

Free and immobilized laccase activities were determined by monitoring the oxidation of 2,2′-azino-bis-(3-ethyl-benzothiazoline-6-sulfonic acid) (ABTS) substrate by laccase, at 30 °C in 0.1 M citrate-phosphate buffer (CPB, pH = 3.0). Particularly, for the measurements of free enzyme activity, the reaction mixture contained 2 mL of an ABTS 240 μM solution, 10 μL of laccase, and CPB (690 μL) up to a total volume of 2.7 mL. For determination of immobilized laccase activity, instead, 3 mg of laccase-immobilized MNPs were put into contact with 2.7 mL of reaction mixture (2 mL of ABTS and 0.7 mL of CPB). The absorbance was measured at 420 nm, and wavelength related to the green-colored cation radical formation ABTS^+∙^ (ε = 36,000 M^−1^cm^−1^) at 30 s time intervals for a total analysis time of 5 min.

The activity of laccase immobilized onto MNPs was normalized with respect to the support weight (specific activity). Particularly, the specific activity was defined as the ratio between the enzyme preparation activity (in units) and support weight:

(3) $$\mathrm{Specific}\mathrm{activity}=\frac{\mathrm{Solid}\mathrm{activity}\left(U\right)}{\mathrm{Support}\mathrm{weight}\left(g\right)}$$

One unit (U) of enzyme activity was defined as the amount of enzyme that catalyzed the oxidation of 1 μmoL of substrate per minute under the experimental conditions. All the experiments were carried out in triplicate.

To verify catalytic performance of laccase bonded to magnetic systems, immobilization efficiency was determined. This parameter was defined as the percentage of solid activity with respect to that deriving from the difference between protein solution activity before and after contact with MNPs (considered as immobilized activity) [21]:

(4) $$\mathrm{Immobilization}\mathrm{efficiency}\;(\%)=\frac{\mathrm{Solid}\mathrm{activity}\left(U\right)}{\mathrm{Immobilized}\mathrm{activity}\left(U\right)}\times 100$$

Additionally, the activity of the two CS-coated MNPs systems, MNPs-CS and MNPs-CS_in situ_, without the immobilized enzyme, was determined in order to verify the influence of the polymer coating on the nanoenzyme activity.

### 2.8. pH, Temperature, and Storage Stability of the Free and Immobilized Laccase

The stability of free and immobilized enzyme was studied by varying some experimental conditions. Specifically, the enzyme activity as a function of pH was determined at 30 °C, in 2.2–6.0 pH range and in 0.1 M citrate-phosphate buffer. In the same buffer, the thermal stability was instead determined by changing temperature in the 25–65 °C range. The storage stability studies were performed at 4 °C in a 0.05 M phosphate buffer (pH = 7.0). The activity assay was carried out at defined time intervals over a 30 day period.

### 2.9. Reusability of Immobilized Laccase

The reusability of the MNPs systems, both containing immobilized laccase and without laccase, was determined by measuring the solid activity after 5 repeated cycles. At the end of each activity assay, the sample was washed with 0.05 M phosphate buffer, pH = 7.0, and a new substrate solution was added.

### 2.10. Removal of a Model Drug

The potential oxidant activity of the developed systems was tested for the degradation of diclofenac (DCF), one of the most common drugs found in water as a pollutant. The degradation studies were performed at room temperature in the presence of ABTS as a mediator. The reaction mixture consisted of 10 μL DCF standard solution (5 mg/mL in methanol), 0.01 M citrate-phosphate buffer pH 3, 1 mL of 270 μM ABTS, and a proper amount of free or immobilized laccase equivalent to 0.02 U of enzymatic activity. Quantitative analysis of DCF transformation was performed by a high-performance liquid chromatographer (Shimadzu, LC-6A, Kyoto, Japan) coupled with a UV–Vis diode array detector (Shimadzu, SPD-M6A). The system was equipped with a reversed-phase column C-18 (250 × 4.6 mm^2^ DI, particle size 5 μm) and a pre-column from Alltech. Injection volume and mobile phase flow were set at 20 μL and 0.6 mL/min, respectively. An isocratic mobile phase containing H_2_O (with 1.3% of formic acid)–MeOH (40:60, *v*/*v*) was used, and the diode array detector was set at 277 nm. Additionally, in this case, the reusability of the systems with immobilized laccase was determined by measuring the solid activity after 5 repeated cycles and compared with that of both MNPs-CS systems without enzymes and free enzymes. Removal efficiency was determined as the percentage of the residual DFC which remained in solution under experimental conditions.

## 3. Results and Discussion

### 3.1. Nanoparticle Characterization

As reported in the Introduction, enzyme immobilization onto magnetic nanoparticles provides many advantages in biotechnological applications with respect to other types of supports. However, to stabilize the immobilized enzyme and to increase the loading capacity of the system, a functionalization of nanoparticles with small molecules or polymers is pivotal. In this study, manganese ferrite nanoparticles (MnFe_2_O_4_), already characterized in a previous study [19], were functionalized by coating them with CS and then used for laccase immobilization. In particular, nanoparticle coating was performed both after MNP synthesis (MNPs-CS) and during their formation (MNPs-CS_in situ_).

#### 3.1.1. Morphological Analysis

**Figure 1:**
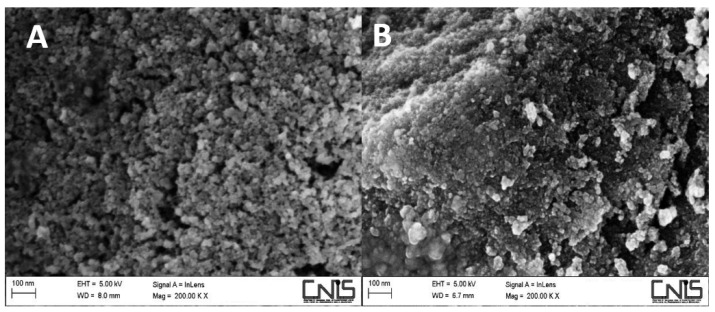
SEM micrographs: (**A**) naked MNPs; and (**B**) MNPs coated with CS during their formation (MNPs-CS_in situ_).

The size and morphology of naked MnFe_2_O_4_ were investigated by SEM analysis, while the CS amount present onto MNPs-CS and MNPs-CS_in situ_ nanoparticles was determined by elemental analysis. SEM measurements evidenced a spherical morphology of the naked MNPs with 15 nm average size (Figure 1A). MNPs size increased up to 20 nm after CS coating (image not shown), and decreased to about 10 nm when chitosan was used during nanoparticle synthesis (MNPs-CS_in situ_, Figure 1B).

#### 3.1.2. FTIR Spectroscopy Analysis

**Figure 2:**
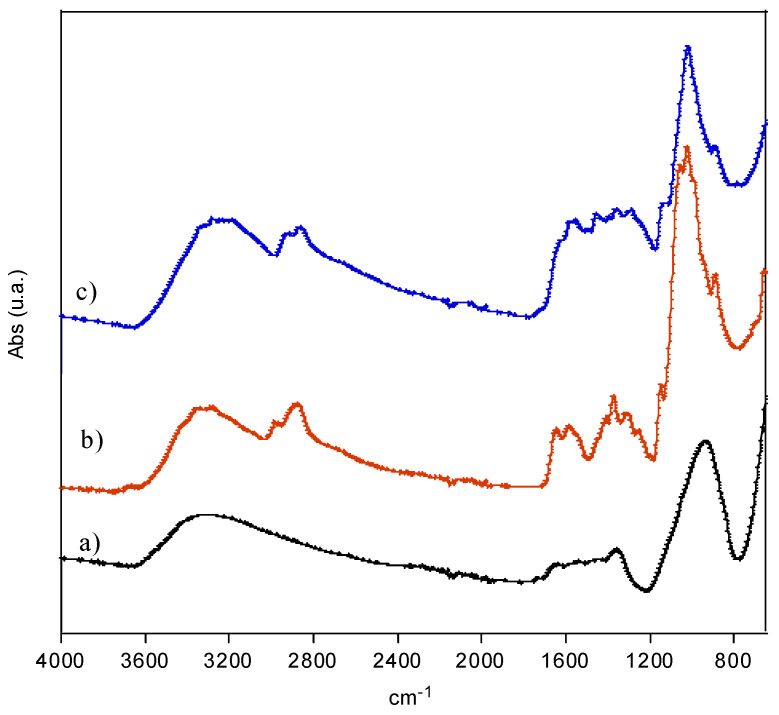
FTIR spectra of the naked MNPs (**a**), CS (**b**), and MNPs-CS nanocomposite (**c**).

FTIR-ATR analysis confirmed the CS coating. In Figure 2, the spectra of MnFe_2_O_4_ MNPs, chitosan, and MNPs-CS are presented. The naked nanoparticles showed characteristic peaks of M–O (metal–oxygen) stretching at 570 cm^−1^, the M–OH vibration in the range 950–1050 cm^−1^, a wide adsorption between 1340 and 1650 cm^−1^ due to both the H–O–H bending (corresponding to water incorporated in the structure) and vibrations attributed to –CH_2_ or –CH groups of the surfactant. Finally, a wide absorption band between 2500 and 3650 cm^−1^ due to the stretching of –OH was evidenced (Figure 2a) [22].

In the spectrum of the MNPs-CS nanocomposite (Figure 2c), the typical absorption bands of chitosan (Figure 2b) were clearly visible. Particularly, the peaks due to the –CH_2_ stretching were observed in the range 2800–3000 cm^−1^, while the amide C=O stretching and the amide NH bending were present at 1650 cm^−1^ and 1550 cm^−1^, respectively. Absorptions in the range 1480–1200 cm^−1^ were attributed to the –CH_2_ and –CH bending, and the wide peak at around 1050 cm^−1^ was attributed to the –C–O–C stretching [23,24].

#### 3.1.3. Elemental Analysis

Elemental analysis confirmed the presence of the organic coating on MNP surfaces (Table 1). For the MNPs-CS sample, data evidenced a polymer content (1%) lower than that of the nanoparticles coated during the synthesis (4%). Probably, in this latter case, the smaller MNP size was responsible for the higher polymer content.

### 3.2. Analysis and Optimization of Laccase-Immobilized Systems

As reported by Wei and Wang, various nanomaterials can possess enzyme-like activity [25]. In particular, Gao et al. first reported that magnetite nanoparticles exhibit an intrinsic peroxidase-like activity [26]. This activity is mainly related to the Fenton-like catalytic activity of iron ions present on the surface of nanoparticles due to the interconversion of ferrous ions (Fe^2+^) into ferric ions (Fe^3+^) in suitable experimental conditions [27].

**Figure 3:**
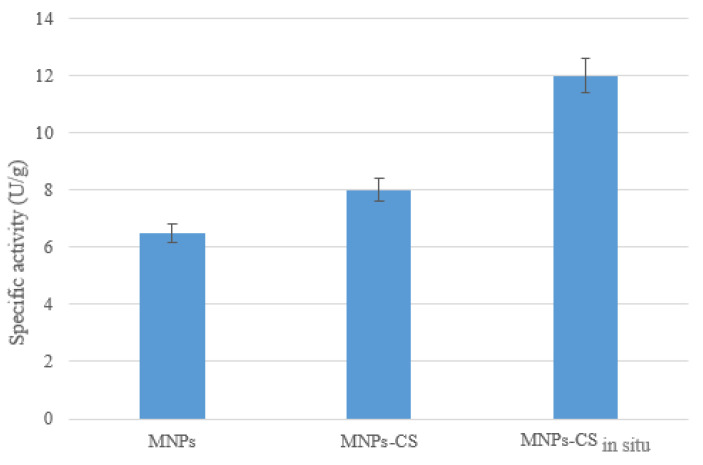
Specific activity of the naked MNPs, CS-coated manganese ferrites (MNPs-CS) and the one coated with CS during their formation (MNPs-CS_in situ_).

These nanoparticles, named nanozyme (nanomaterial-based artificial enzymes), have received great attention, and, to date, many studies have emerged in this field. As reported by Mumtaz et al., this category includes metallic and bimetallic nanostructures (Au, Pt, Au/Ag), metal oxide nanoparticles (CeO_2_, V_2_O_5_, CuO, MnO_2_), metallic sulfides (FeS, Fe_3_O_4_, CuS), metal–organic frameworks (MIL-53(Fe), MIL-68), carbon-based nanomaterial (graphene oxide, carbon nanotubes), and hybrid materials (graphene oxide–Au, Pd/Fe_3_O_4_–PEI–graphene oxide) [28]. Nanozymes have been used as alternative systems for a broad range of applications thanks to their high stability and ease of functionalization [29]. Indeed, they are able to act on common substrates of many enzymes—typically, oxidoreductase and hydrolases—and for this reason, they have been widely utilized for environmental treatments, biosensing, immunoassays, and disease therapy [30]. As for the MnFe_2_O_4_ nanoparticles synthesized in this work, it was found that the naked MNPs exhibited a not Fenton-type intrinsic-specific activity against ABTS of about 6.5 U/g (Figure 3), attributed to Fe^3+^ ion reduction. After coating with the polysaccharide, the MNPs-CS system showed a slight increase in catalytic response (~8 U/g). These data suggested a contribution of CS to the catalytic activity of the MnFe_2_O_4_ nanoparticles. Probably, owing to the polycation features, CS could promote the interaction of MNPs with the negatively charged substrate and, afterwards, ABTS could diffuse towards Fe^3+^ surface ions thanks to the polymer swelling.

The MNPs-CS_in situ_ nanoparticles, obtained by replacing NaDBS surfactant with chitosan during the synthesis, showed a specific activity of 12 U/g higher than the MNPs-CS system (Figure 3). Probably, the higher polymer amount present on the MNPs-CS_in situ_ nanocomposite surface allowed a greater supply of the substrate and then a higher redox activity of the support.

Thereafter, laccase was covalently bonded onto CS-coated MNPs after nanoparticles’ activation with glutaraldehyde. In the literature, various studies have investigated and elucidated the role of the support on the immobilization of laccase from different sources. However, to the best of our knowledge, little attention has been paid to evaluating the coating influence on the loading and specific activity of *Trametes versicolor* laccase immobilized on magnetic nanoparticles, or on the magnetic support contribution to the catalytic activity of systems.

According to data in the literature, a relationship between enzymatic activity and the amount of glutaraldehyde used for the activation of MNPs has been found. Particularly, the enzyme activity increases with increasing glutaraldehyde concentration, showing a maximum value at ca. 1% (*v*/*v*) concentration [31]. For this study, we chose such a concentration.

**Figure 4:**
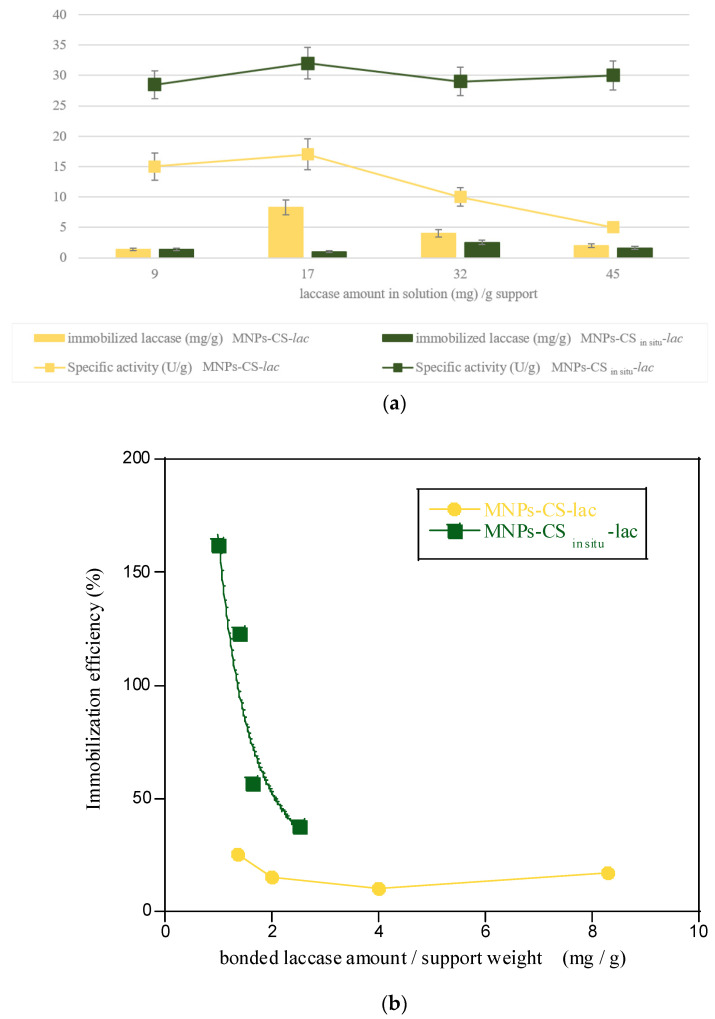
Immobilized laccase onto magnetic systems in the function of enzyme amount in solution normalized per support weight and respective specific activities (**a**); immobilization efficiency vs. laccase amount bonded onto magnetic systems (**b**).

To verify the influence of laccase concentration on enzyme loading, enzyme concentration was varied in the range 0.20–1 mg/mL, corresponding to 9–45 mg protein/g support. The obtained results are reported in Figure 4a. The best results were achieved at the enzyme concentration of 0.37 mg/mL (17 mg protein/g support) and 0.72 mg/mL (32 mg protein/g support). In particular, the immobilized enzyme amount was 8.3 mg of enzyme/g for MNPs-CS-*lac* (immobilization yield = 49%) and 2.5 mg of enzyme/g for MNPs-CS_in situ_-*lac* (immobilization yield = 8%). However, except for the 0.2 mg/mL concentration, the amount of enzyme immobilized on the support coated with CS after synthesis (MNPs-CS-*lac*) was higher than that bonded on the support coated during synthesis (MNPs-CS_in situ_-*lac*). This behavior could be related to the greater polymer concentration present on the MNPs-CS_in situ_ system that probably favors nanoparticle aggregation, making binding sites for laccase less available. Moreover, generally, at higher protein concentration, the immobilization yield decreased for both developed systems, probably due to the formation of laccase aggregates, which hinder protein diffusion towards the supports. Indeed, the affinity between the cationic support and laccase, having a negative net charge, is mainly due to electrostatic interactions, which may be compromised by the formation of enzyme aggregates. In fungal laccases, at high enzyme concentration, existence of aggregate forms has been verified with a consequent reduction in active catalytic sites. Such aggregates can be associated to the enzyme “resting” form [32]. On the contrary, as for the immobilized laccase-specific activity, values around 30 U/g were found for the MNPs-CS_in situ_-*lac* system, higher than those determined for MNPs-CS-*lac* (Figure 4a). Overall, the specific activities found in this study are lower than those reported in the literature. Indeed, high enzyme activities were reported for laccases from *Trametes versicolor* and *Echinodontium taxodii* immobilized onto Fe_2_O_3_/SiO_2_ (224U) [33] and PEG–Fe_2_O_3_ (849 U/g) [34] supports, corresponding to higher amounts of protein immobilized onto solids (62.2 and 24.4 mg/g, respectively). However, the contribution of the nanoparticles alone to the catalytic activity of the systems was not studied.

**Figure 5:**
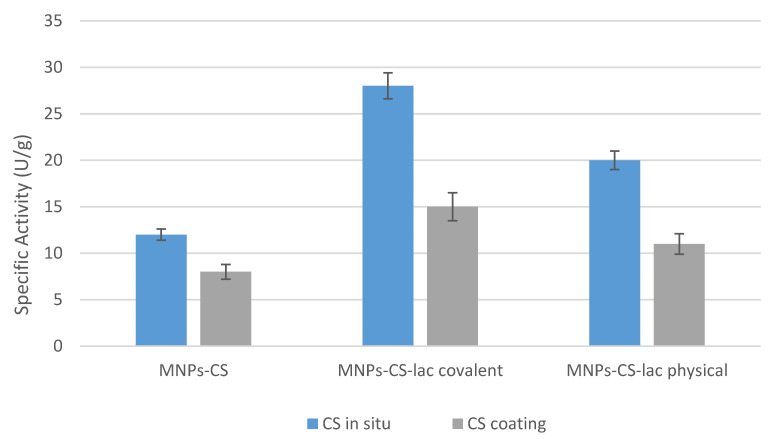
Specific activity of the two MNPs-CS systems containing covalently and physically immobilized laccase compared with that of the MNPs-CS system without enzymes.

In Figure 4b, immobilization efficiency vs. bonded laccase amount normalized per support weight was reported. According to obtained specific activity data, it was confirmed that the MNPs-CS_in situ_-*lac* system possessed the best performances. In addition, at low bonded enzyme amount efficiency, values higher than 100% were observed. These findings highlighted the contribution of coated support to catalytic activity (Figure 5).

To verify if the covalent bond could affect the enzyme activity, laccase was also physically adsorbed on the supports in the absence of glutaraldehyde. Physical adsorption was performed by using an enzyme concentration of 0.2 mg/mL, because the catalytic activity of the systems with covalent laccase obtained at this binding concentration was similar to that obtained at a 0.37 mg/mL enzyme concentration. Immobilized laccase amounts of 1.5 and 1.8 mg/g were found for MNPs-CS-*lac* and MNPs-CS_in situ_-*lac*, respectively. The specific activity of such systems having physically adsorbed laccase was compared with that of MNPs coated with CS without enzyme or with covalently bonded enzyme, according to the two coating procedures, CS coating or CS in situ (Figure 5).

Generally, the MNPs-CS_in situ_ supports with or without laccase showed a specific activity higher than that of MNPs-CS. Presumably, the greater amount of CS present on the MNPs-CS_in situ_ system, as evidenced by elemental analysis (Table 1), allowed the support to contribute significantly to the specific activity of the system, as previously explained.

In addition, the physically adsorbed laccase on both supports (MNPs-CS-*lac* physical) possessed a catalytic specific activity lower than that observed for the covalently immobilized enzyme (MNPs-CS-*lac* covalent). Probably, laccase was immobilized in a more active conformation when bonded to the supports.

The immobilization procedures of enzymes onto solid supports can provoke important structural changes that affect the stability and activity of the bonded proteins. Different spectroscopic techniques can be used to monitor structural, conformational, and electronic variations of immobilized enzymes [35]. Generally, chemical immobilization leads to a reduction in enzyme activity. However, the orientation of the protein active site on the support surface is the most important parameter influencing the activity. In our case, covalent laccase immobilization onto the coated magnetic supports probably led to an orientation of the enzyme active site more suitable for substrate bonding.

### 3.3. Bioreactor Characterization

The developed systems were characterized in terms of stability of pH, temperature, and time. In addition, the possibility of their reuse was evaluated. As for stability of the systems at pH variation, in agreement with the literature [36,37], the best laccase catalytic activity was found at pH = 3 both in free and immobilized forms (data not shown). As for thermal stability, generally, the enzyme immobilization on a support stabilized the biocatalyst against temperature variation. Kalkan, Nuzhet Ayca et al. observed that systems containing immobilized laccase possessed activity higher than free laccase in the range of 10–40 °C [38].

**Figure 6:**
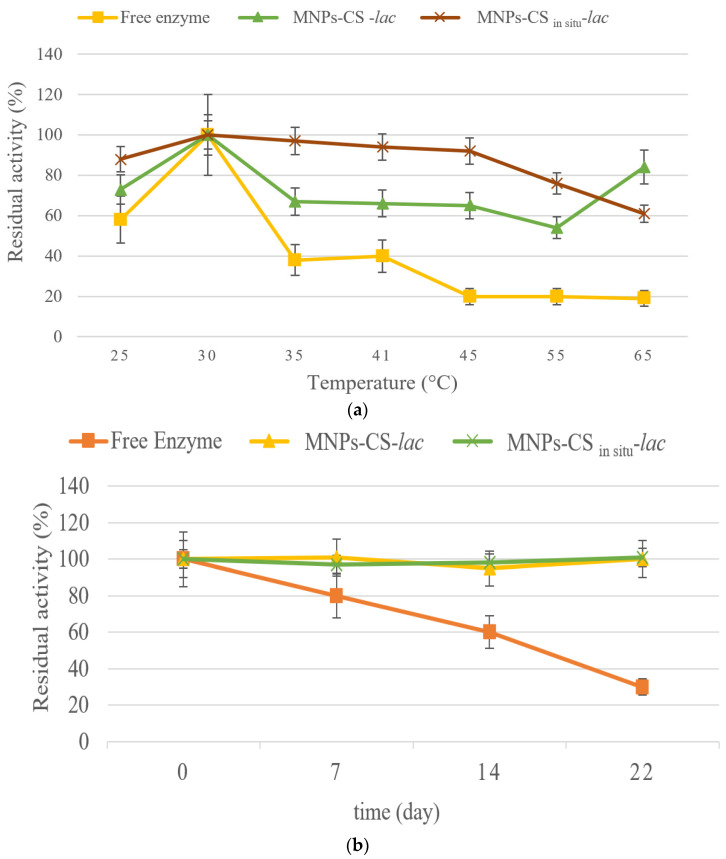
Residual activity as a function of temperature (**a**) and time stability (**b**) of free and covalently immobilized laccase onto MNPs-CS and MNPs-CS_in situ_ systems.

In Figure 6, the residual activity of laccase as a function of temperature (Figure 6a) and storage time (Figure 6b) is reported. As it can be observed, the enzyme showed the maximum activity at 30 °C, both when free and immobilized. However, the stability of free laccase decreased drastically when the temperature was higher than 30 °C. In contrast, a good resistance to temperature denaturation was evidenced for the immobilized enzyme, particularly when the MNPs-CS_in situ_ support was used for immobilization. However, in both systems, a residual activity of about 60% up to 65 °C was observed. As for time stability, free laccase lost activity over time, retaining only 28% of residual activity after 20 storage days (Figure 6b). In contrast, the enzyme immobilized onto the two CS-coated MNPs displayed remarkable stability for 30 days. These results are in accordance with data in the literature, showing that after 40 days, laccase immobilized on ferrite nanoparticles preserved a residual activity about two-fold higher (89%) than the free enzyme (49%), thus confirming the advantages of enzymatic immobilization [39].

**Figure 7:**
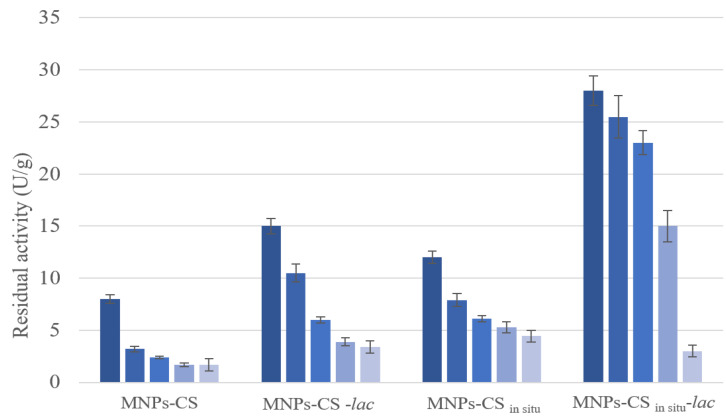
Reuse cycles in the oxidation reaction of ABTS by the magnetic systems without and containing immobilized laccase.

Finally, operational stability of the developed MNPs containing covalently immobilized laccase, compared with that of MNPs without the enzyme, was evaluated in a repeated batch process (five reuse cycles, Figure 7). The MNPs-CS-*lac* and MNPs-CS_in situ_-*lac* systems showed a catalytic activity higher than CS-coated MNP systems without enzymes, thus evidencing the contribution of the enzyme, particularly for MNPs-CS_in situ_-*lac*. At the third cycle, the enzyme activity of MNPs-CS-*lac* and MNPs-CS_in situ_-*lac* was 2.5-fold and 3.8-fold higher than MNPs-CS and MNPs_CS_in situ_ (without enzyme), respectively. However, for the MNPs-CS_in situ_-*lac* system, the residual activity result was more persistent, only showing a reduction of about 50% of the catalytic activity at the fourth cycle. Unfortunately, given the small size of the MNPs and their good dispersion in aqueous buffered medium, a loss of material after some recycling was noted. Therefore, the considerable loss of activity after the third cycle could also be attributed to the difficult recovery of the material after several reuses.

However, our results showed that the CS-coated MnFe_2_O_4_ magnetic nanoparticles can be promising supports for laccase immobilization, not only for the improved thermal and storage stability, but also for the good contribution to the system’s catalytic activity of the polymer coating, a very important feature in the case of enzyme denaturation or detachment.

### 3.4. Diclofenac Degradation

It has been widely reported that laccase, both free and immobilized, can be an efficient material for the removal of pollutants [40]. Thus, the potential applicability of the developed MNPs-CS systems containing immobilized laccase was assessed towards the degradation of diclofenac (DFC), the most common non-steroidal anti-inflammatory drug. The catalytic activity of laccase towards this class of compounds has been shown to be reduced in the absence of an appropriate mediator; therefore, the removal reaction was carried out in the presence of ABTS.

**Figure 8:**
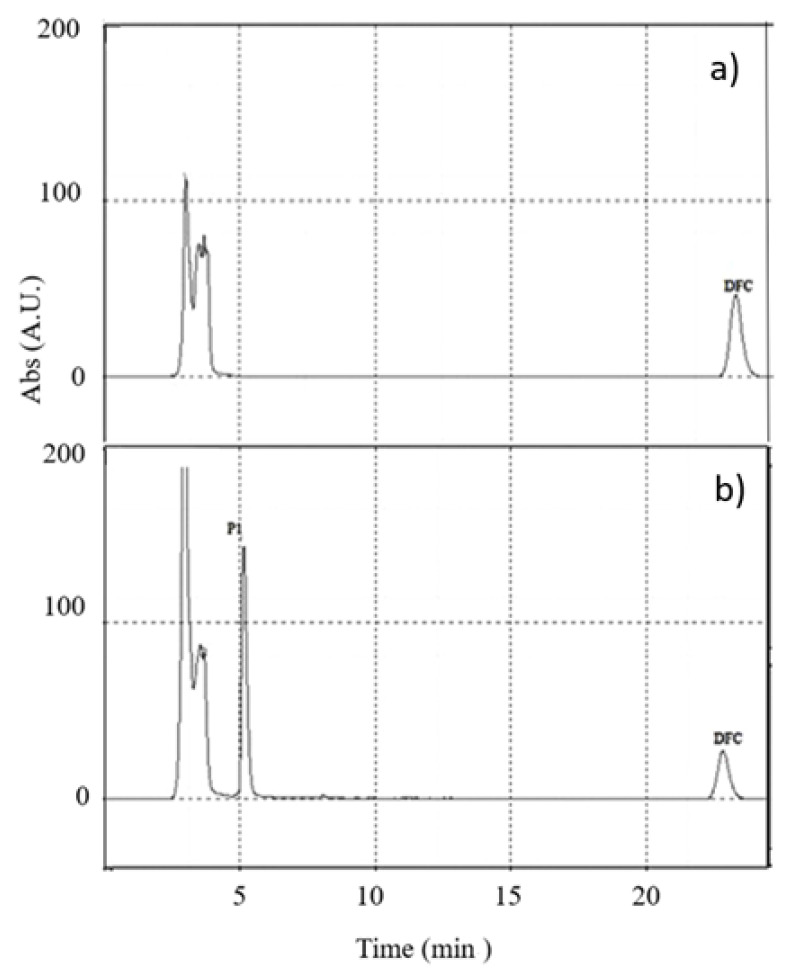
Chromatograms of diclofenac before (**a**) and after 30 min treatment with free laccase (**b**).

High-performance liquid chromatography (HPLC) enabled identification of the drug at every stage of its removal. Diclofenac was eluted at a retention time of 22 min (Figure 8a). Treatment with free laccase caused an evident decrease in the well-resolved peak, with concomitant formation of a degradation product (P1) (Figure 8b).

**Figure 9:**
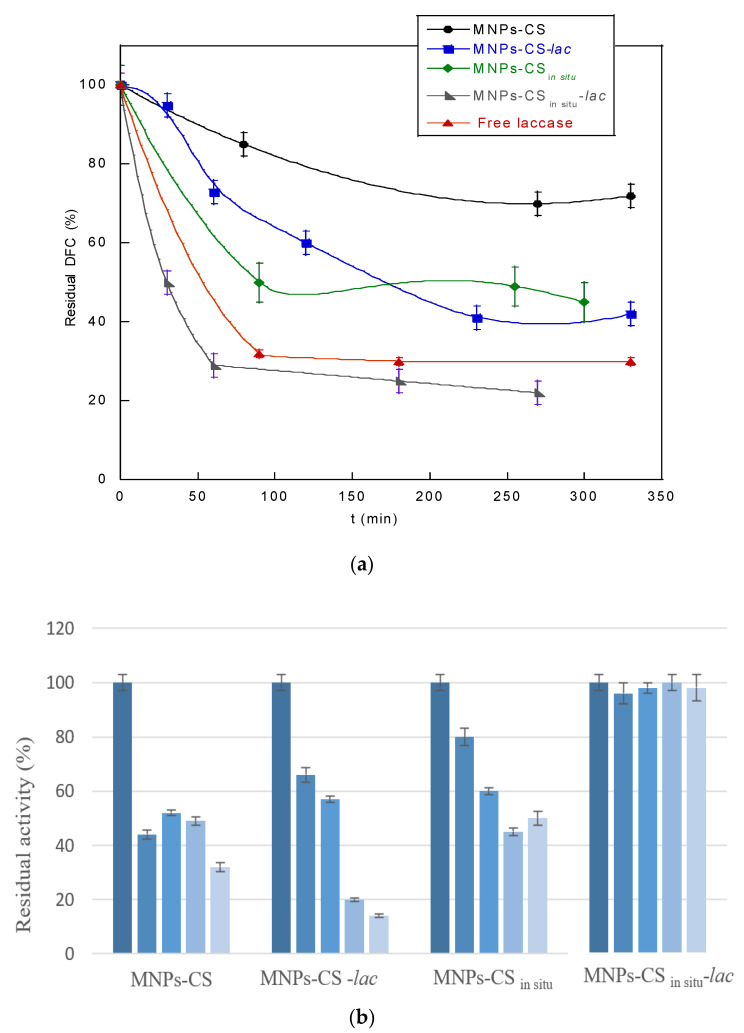
Residual percentage of diclofenac (DFC) after treatment with the developed magnetic systems and free laccase (**a**); reuse cycles in DFC removal by the magnetic systems (**b**).

The time-course removal of diclofenac by the developed immobilized supports compared with the free enzyme is reported in Figure 9a. It was observed that all the systems possessed a catalytic action towards the drug, but with different degradation rates. The systems containing the immobilized enzyme showed a degradation higher than that of the correspondent controls (systems without laccase). In particular, the MNPs-CS-*lac* and MNPs-CS_in situ_-*lac* supports removed 60% and 78% of DFC, respectively, at 270 min. In addition, the MNPs-CS_in situ_-*lac* system was more efficient than the free enzyme (70% drug removal). Probably, the active conformation assumed by the enzyme after covalent immobilization together with the contribution to the catalytic activity of the polymer-coated magnetic support could justify the observed difference in DFC degradation both in terms of removal efficiency and the kinetics of degradation. The reuse of solid biocatalysts plays a key role in pollutant removal. In this case, compared with the free enzyme, the magnetic biocatalyst can be easily separated from the reaction medium by an external magnetic field and reused. The good removal efficiency of the MNPs-CS_in situ_-*lac* system was further verified by carrying out five cycles of DFC degradation (Figure 8b). The obtained findings showed that the best removal efficiency and reuse were obtained when the enzyme was immobilized on magnetic nanoparticles coated with CS during the synthesis phase. Specifically, almost no decrease in the catalytic activity was evidenced for MNPs-CS_in situ_-*lac* up to the fifth cycle.

## 4. Conclusions

Manganese iron oxide nanoparticles (MNPs) were synthesized by a reverse micro-emulsion method and used as supports for the covalent immobilization of laccase from *Trametes versicolor*. It was found that the naked MNPs exhibited a not-Fenton-type intrinsic activity against ABTS, attributed to the Fe^3+^ surface ions. To avoid nanoparticle aggregation and to bind laccase on MNPs more effectively, chitosan was employed as a polymer coating and glutaraldehyde as a crosslinking agent. Polymer coating was carried out both after (MNPs-CS) and during (MNPs-CS_in situ_) MNP synthesis. It was verified that both CS-coated supports possessed a specific activity more than the naked MNPs. Such intrinsic activity, related to the CS cationic and hydrophilic features promoting both MNPs/ABTS interactions and diffusion of the ABTS towards Fe^3+^ ions, was higher for the MNPs-CS_in situ_ system because it contained greater CS amounts. An enhancement of the specific activity of the systems was evidenced when laccase was immobilized either physically or covalently.

However, the physically adsorbed enzyme possessed a specific activity lower than covalently immobilized laccase, suggesting that the covalent bond enabled enzyme immobilization in a more active conformation, especially for the MNPs-CS_in situ_-*lac* system. The immobilization efficiency values also confirmed the best performance of this system.

Notwithstanding the pure magnetic supports (without enzyme) which showed a certain catalytic activity, a significant enhancement of the specific activity of the systems was evidenced when laccase was covalently immobilized.

Contrary to the free enzyme, the MNPs-CS_in situ_-*lac* and MNPs-CS-*lac* systems showed both a good resistance to temperature denaturation and storage stability for 30 days. When applied for the removal of DFC, the most promising system was MNPs-CS_in situ_-*lac*, which showed a removal efficiency of 78%, higher than the free enzyme (70%). The good drug removal efficiency shown by the MNPs-CS_in situ_-*lac* system was also confirmed by subjecting the system to five cycles of reuse. Almost no decrease in the catalytic activity was highlighted for this system, thanks to the active conformation assumed by the covalently immobilized enzyme and to the contribution to the activity of the CS-coated support. This study showed that the developed MNPs-CS _in situ_-*lac* system could be an excellent candidate for the removal of contaminants.

## Figures and Tables

**Table 1:** Elemental analysis of uncoated MnFe_2_O_4_ (MNPs), CS-coated MNPs (MNPs-CS and MNPs-CS_in situ_ samples), and CS.

Sample	C (%)	H (%)	N (%)	Polymer Amount (%) *
CS	45	7	8	-
MNPs-CS	0.5	0.4	0.2	1
MNPs-CS _in situ_	1.8	1.0	0.9	4

* The coating content was determined by the ratio between C content (%) in the CS-coated MNPs and that in the pristine polymer.
